# Supplementary material for: Novel insights into immune stress markers associated with myxosporeans gill infection in Nile tilapia (molecular and immunohistochemical studies)
Source: PLoS One. 2024 Jun 4;19(6):e0303702. doi: 10.1371/journal.pone.0303702 (PMC11149867; doi:10.1371/journal.pone.0303702)
Supplement: Attachment — Submitted filename: reviewer report for PLOS one 15-3-2024.docx [file pone.0303702.s001.docx]

The study conducted by Ramadan et al. is very interesting and will be useful for PLOS one readers. The manuscript the hypothesis of the study is clear and the manuscript is well-written. Minor issues are needed for improving the manuscript before being published.

**Abstract**

1-the abstract should be informative, all genes abbreviations should be mentioned in full formula for the first time, then use the abbreviated form

2- the authors should mention the methodology in brief (I mean, no of fish used in the study, no of groups, no of replicates, duration of the study).

3- The abbreviated form of genes should be italic

4-line 69, the abbreviations should be written in full formula

5-line 71, the abbreviated gene name should be italic

6-line 98, *Oreochromis niloticus* should replace by *O. niloticus*

7-line 153, µl should be replaced by µL and should be revised in all manuscript.

8-line 179, remove (,)

9-line 182, add the name of anti-coagulant

10-190, which organs were sampled and no of samples

11-line 202, what about normality test?
